# SOCIAL SUPPORT DYNAMICS FOR SOUTH ASIAN BREAST CANCER PATIENTS: AN ANALYSIS CONDUCTED USING ATLAS CAREMAPS

**DOI:** 10.1093/geroni/igac059.2238

**Published:** 2022-12-20

**Authors:** Ranak Trivedi, Ambri Pukhraj, Shreya Desai, Rishabh Shah, Rashmi Risbud, Lidia Schapira, Dolores Gallagher-Thompson, Karl Lorenz

**Affiliations:** Stanford University, Palo Alto, California, United States; Stanford University, Stanford, California, United States; Veterans Affairs Palo Alto Health Care System, Menlo Park, California, United States; Stanford University, Stanford, California, United States; University of California, Davis, Davis, California, United States; Stanford University, Stanford, California, United States; Stanford University, Stanford, California, United States; Veteran Affairs Palo Alto Health Care System, Stanford, California, United States

## Abstract

With a rise in the prevalence and a drop in mortality rates of breast cancer among South Asians (people with heritage from India, Pakistan, Nepal, Bhutan, Sri Lanka, Maldives, and Bangladesh), globally and in the US, there is an increasing number of South Asians managing breast cancer. The South Asian Family Approaches to Disease (SAFAD) study aims to better understand how South Asian breast cancer survivors are supported while managing breast cancer. We conducted semi-structured interviews to complete an adapted version of Atlas CareMaps, a visual representation of survivors' care networks at the time of the interview. Thirteen South Asian breast cancer survivors were enrolled. Survivors were on average 47y (SD=9.1y) and reported being diagnosed with stage 0 (n=1), stage 1 (n=3), stage 2 (n=6), or stage 4 (n=3) breast cancer. Analyses of the Atlas CareMaps suggest 1) South Asian breast cancer survivors received support from 13.7±3.5 individuals, while providing care to 3.3 ± 2.2 individuals; 2) at more advanced stages of breast cancer, patients provide less support to others (Stage 1=3.8±2.2, Stage 4=1.7±1.5); 3) older survivors received more support from abroad (< 40y=2.0±2.2, patients 50+=5.3±3.3). Atlas CareMaps can provide useful insights into the rich care networks of South Asian breast cancer survivors which can be used to develop clinical programs.

